# Use of community treatment orders in an inner-London assertive outreach service

**DOI:** 10.1192/pb.bp.112.042184

**Published:** 2014-02

**Authors:** Muffazal Rawala, Susham Gupta

**Affiliations:** 1 South London and Maudsley NHS Foundation Trust, London; 2 East London NHS Foundation Trust, London

## Abstract

**Aims and method** To compare admission rates and bed occupancy before and after the introduction of community treatment orders (CTOs) in 37 assertive outreach service patients. The effect of CTOs on treatment adherence and illicit drug use were also evaluated. The views of patients and care coordinators were obtained through a focus group.

**Results** When CTOs were introduced, admission rates fell from 3.3 to 0.3 per year and average bed occupancy declined from 133.2 to 10.8 days per year. Treatment adherence improved from 4 (10.8%) to 31 (83.7%) patients, and an objective reduction in substance misuse was observed in 25 (67.5%) patients. Whereas patients expressed ambivalence towards CTOs, their care coordinators generally had a more positive view.

**Clinical implications** The decline in hospital usage following the introduction of CTOs is encouraging and could reflect improved adherence and engagement through intensive case management, leading to a reduction in readmissions. However, further studies need to look at quality of life, cost-effectiveness and the impact on patients.

With the focus of psychiatric care now firmly in the community there has been a steady reduction of costly in-patient beds along with the emergence of the community recovery model.^1,2^ The benchmark of successful patient management now includes reduced hospital usage. The management of psychiatric patients in the community enables greater patient autonomy. This, however, also results in some patients disengaging and relapsing, leading to repeated admissions with poorer clinical outcomes as well as greater financial stress on the healthcare system. In addition, the media's portrayal of psychiatric patients as dangerous led to a call for greater scrutiny of community care under legal jurisdiction by the political establishment.^3,4^ In 1998 the government started the process of reforming the mental health law in England and Wales resulting in amendments to the Mental Health Act 1983 and introducing supervised community treatment (SCT) orders – or community treatment orders (CTOs) as they are more commonly known – in 2008. The initial predictions of the government that CTOs would be used only for a small number of ‘revolving-door patients’ was a vast underestimation.^5^

Given there are over 10 000 people with CTOs^5^ their impact needs to be better understood. A recent randomised controlled trial^6^ in England failed to find any benefit between those assigned to Section 17 leave *v.* CTOs. However, they did not compare the outcomes of community mental health team (CMHT) and assertive outreach team (AOT) patients.^6^ The aim of our study was therefore to identify the impact of CTOs on an AOT population in inner London, in terms of admission rates and bed occupancy – proxy markers for relapse prevention and morbidity respectively. The paper also attempts to capture some of the views of patients with CTOs and that of their care coordinators.

## Method

This study is a naturalistic, observational, retrospective, before-and-after analysis of patients on CTOs, looking at its impact on proxy indicators of clinical outcomes. The study was carried out in a City and Hackney Assertive Outreach Service (CHAOS) in the inner-city Borough of Hackney, East London. The referral criteria for patients to the AOT include a diagnosis of a primary psychotic illness and a history of significant disengagement, non-adherence to treatment and recent history of repeated hospital admissions. All patients were over the age of 18 years and without a primary diagnosis of an organic brain injury or of substance misuse. In this study each patient served as their own comparator for the annual average admission rate and bed occupancy for the 2-year pre-CTO period compared with the CTO period. The inclusion criteria were (a) all patients under the care of the AOT who have a CTO within the study period of November 2008 to April 2012, and (b) must have been on the CTO for a minimum period of at least 6 months. Patients on a CTO for less than 6 months were excluded as it was felt that the duration may be too short to have a definitive impact on clinical outcome measures. Ethical approval was granted by the East London NHS Foundation Trust ethics subcommittee as part of a service evaluation to look at the impact of CTOs on the clinical outcomes of AOT patients.

The primary outcome measures studied were number of admissions per year and bed occupancy (days per year) both in a 2-year period prior to being on a CTO (pre-CTO period) compared with that of the CTO period. The amount of overnight leave that patients utilised under Section 17 leave was subtracted from the duration of admission, hence giving an accurate account of bed occupancy. Secondary outcome measures included difference in adherence to psychotropic medication and the level of illicit drug use during these two periods. The effect of a CTO on a patient's quality of life was also assessed under the domains of stable housing, financial stability, social support system and engagement in vocational activities. Data were collected from the electronic patient records system (RiO) used by the local services. Admission and bed-occupancy rates were collected from hospital admission data. Substance misuse and adherence to prescribed treatment were elicited from records for the 3-monthly medical reviews and care plan approach (CPA) document and included results of urine drug screens.

The second part of the study attempted to elicit the views of the patients and their care coordinators about the impact of CTOs. Support workers who were not directly involved in decision-making regarding CTOs were used to interview the patient to reduce reporting bias. Getting the patient's perspective is already part of the standard routine care.

## Results

A total of 37 patients were identified in the CHAOS caseload who had been subject to a CTO for a minimum of 6 months during the study period. This was deemed an adequate sample size and study period (40.7 patient years on a CTO compared with 74 pre-CTO patient years) to meaningfully measure any impact of the intervention, especially in the absence of such studies in the UK. The mean duration of CTOs in this patient group was 13.2 months (range 6–28). The average age was 40.9 years (range 25–65); and 34 of the 37 patients were men (92%). All the patients had a primary diagnosis of a psychotic disorder. Paranoid schizophrenia was the most common Axis I diagnosis (*n* = 22, 59.5%), followed by bipolar affective disorder (*n* = 10, 27%) and schizoaffective disorder (*n* = 5, 13.5%).

The mean admission rate was 3.3 admissions per year (range 1–6) for the pre-CTO years and this declined to 0.3 (range 0–2) during the CTO phase – a fall of 90%. A total of 26 patients did not have any hospital admission during the CTO periods, whereas 11 were recalled to hospital during this time. The average bed occupancy during the pre-CTO period was 133.2 days per year (range 40–365) compared with 10.8 per year during the CTO period, a decline of 92%. The average duration of hospital admissions consequently fell from 40.4 days per individual admission to 36.4 days, a slight reduction of 4 days (10%) per admission.

Non-adherence with psychotropic medication was observed in 31 (83.7%) patients in the pre-CTO period, whereas only 4 (10.8%) patients were non-adherent during the CTO period. In total, 17 (46%) patients were on depot medication and the rest were on oral alternatives. Adherence was measured by documented evidence of administration of depot antipsychotics by the team (17 individuals), attendance at a clozapine clinic and plasma clozapine blood levels (8 individuals) and supervised oral antipsychotic (other than clozapine) medication (12 individuals). In 12 of the 20 patients on oral medication there was evidence of attempts at progressive transfer of responsibility and self-medication with increasing insight and motivation. For these 12 patients collection of repeated prescriptions was used as a measure of adherence.

In the pre-CTO period 57% of the patients (*n* = 21) were documented to have been using illicit drugs. The most common drug used was cannabis (27%, *n* = 10), followed by cocaine (16.2%, *n* = 6) and heroin (2.7% *n* = 1), and 10.8% (*n* = 4) had used more than one illicit drug. Of the cohorts who were using drugs before commencement of a CTO, 67.5% (*n* = 25) had objectively stopped using during the CTO period. All patients had contact with a dedicated dual-diagnosis worker within the AOT, as part of their care plans all patients had both random and scheduled urine drug screens prior to their CPA meeting. Quality of life was assessed under the domains of stable housing, financial stability, social support system and engagement in vocational activities; 78.4% of patients (29 out of 37) showed an improved quality of life in the CTO period.

### Patients' and care coordinators' perspectives on CTOs

The views of patients were explored in a focus group and facilitated by the support workers with the AOT. Participation rate was low, with only six patients attending the session (Box 1). One of the researchers (M.R.) approached the care coordinators of each patient for their views on the clinical impact of CTOs (Box 2). It is evident that the patients' views were mixed. Although not representative of the entire patient group it is clear that CTOs are largely considered by patients to be restrictive, despite their objective clinical improvement. The contrast between the views of the patients and the care coordinators points to the difference in perspectives on the use of CTOs in the community.

## Discussion

### A brief history of CTOs

The deinstitutionalisation of psychiatric services in the Western world, which began in the 1960s, has led to improved community care provision. However, this has also resulted in some patients having frequent readmissions.^7^ In North America, CTOs were established in the 1980s to address this problem. Their introduction was thought to offer an ethical alternative to managing at-risk individuals that was less restrictive than formal hospital admissions. Over the past decade there has been a shift in the way CTOs are viewed, from being a tool to manage risk behaviour to being an important treatment intervention that can help prevent relapses, reduce in-patient admission days and possibly lead to higher functioning.^8^ Until recently, CTOs were used primarily in North American^8^ and Australasia,^9^ but Scotland, England and Wales, and Israel have now introduced CTOs into their mental health legal framework. However, cross-national comparisons are difficult because of differences in the structure of community mental health services, the legislation and criteria for implementing them.^10^

**Box 1** Patients' views of community treatment orders (CTOs)

Effects of CTO on mental health

‘It [CTO] was helpful being looked after, as more therapeutic input.’

‘Being in hospital could be good; anytime I become unwell, I was admitted directly, very good that was’.

‘No, I am already good. I do not need treatment.’

On conditions of a CTO

‘Took it as not an obligation but an activity that I could comply with and keep healthy.’

‘I am not a prisoner; you people do not leave me alone.’

‘Made me feel accomplished, something to look forward to.’

Adherence

‘Yes, but I did not like being monitored. Felt pressured, they should just trust me. I like doing it my way.’

‘I am not unwell.’

‘I am not unwell, psychiatric medicines can control people.’

Alternatives to the restrictions of CTO

‘You should focus more on individual talk therapy. Everything is not your medicines.’

‘If I had good social support and a relationship that would also act as a CTO.’

‘People should just carry on with their lives. I make my own rules.’

**Box 2** Care coordinators' views of community treatment orders (CTOs)

‘The power of recall seems particularly important with this client. So far there has been a picture of rapid relapse despite being in a staffed setting.’

‘CTO has been used for this client in the hope that it provides a supportive framework.’

‘CTO has helped to improve compliance and engagement with all of my patients.’

‘CTO appears to work with clients who believe in them and respect boundaries.’

‘CTO essential for this very difficult man to give him structure and compel him to take medication.’

‘CTO could lead to increase of mistrust especially if patient does not have good understanding of their illness and not willing to be involved with mental health services.’

‘It has reduced my client's drug use and repeated admissions.’

‘Is it ethical to impose restrictions in the community? Still, it has helped some patients engage and take treatment.’

‘It helps keep my patients out of hospital but lots of paperwork.’

In the USA, CTOs are divided into ‘least restrictive’ and ‘preventive’. The former provides for a least restrictive alternative to hospital treatment. The preventive model enables treatment of deteriorating mental state to prevent dangerousness. The current US trend has been to move towards a preventive CTO model because of difficulties with the design model of least restrictive CTOs. In parts of Canada the preventive CTO model also incorporated ‘consent criteria’.^11^ The CTO model in Australasia, Israel and Scotland has incorporated both features of the least restrictive and preventive models. These countries exclude the essential North American criteria of ‘imminent dangerousness’, leading to a much broader criteria for community orders.

Scotland introduced Europe's first CTO in October 2005. The criteria for the Scottish CTO include risk management, a focus on prevention and modified incapacity criteria worded as ‘significant impairment of decision making’.^12^ Prior to the introduction of CTOs in England and Wales in 2008, compulsory community treatment existed in various guises in the Mental Health Act 1983. These included Section 17 for conditional leave from hospital, with patients being sent on extended periods of leave. The Act also made provisions for statutory guardianship under Section 7 in the community for patients who needed a legal safety framework for maintaining conditions of residence but who were willing to cooperate and abide by their treatment plan and did not require mandatory community treatment. For forensic patients there has been provision of SCT in the form of Section 37/41; once the patient is ready to be discharged from in-patient services, the Secretary of State by virtue of the restriction order, Section 41, has to agree and attach conditions to the discharge from hospital. Supervised discharge orders were introduced by the Mental Health (Patients in the Community) Act 1995 to ensure community aftercare provisions for patients with serious mental illness who were detained in hospital. They were primarily used as risk management tools with little leverage for enforcing appropriate treatment adherence.

The use of CTOs in England and Wales was intended to enable patients who no longer need to be detained in hospital to live in the community under the powers of the Mental Health Act.^13^ Patients detained under Section 3 or subject to certain specified provisions under Part III of the Act (namely a hospital order, a hospital direction or a transfer direction without restrictions) could be considered for a CTO to prevent their deterioration on discharge from hospital through improved adherence with medication in the least restrictive manner. The Department of Health^14^ stated that the introduction of CTOs was aimed at helping patients engage based on the recovery model and to manage the risk some patients may pose to themselves and others. Despite the absence of clear evidence of their effectiveness, the ethical debate about their use and opposition from patients, mental health charities and the civil rights lobby, CTOs were formally introduced in England and Wales in 2008.

A King's Fund paper^15^ in 2005 predicted that the use of CTOs in England would be between 2 and 50 per 100 000 of the general population. The government grossly underestimated the clinical and risk management appeal of CTOs and only expected a few hundred such orders. However, by 2011, the number of CTOs had reached an estimated 10 000 in England and Wales.^5^ There has been strong support for their use from service providers.^16^ Among UK psychiatrists the potential for treatment adherence, authority to treat the patient and ensuring early identification of relapse have been key points in deciding on a CTO for a patient.^16,17^

### Assertive outreach teams and CTOs

Assertive outreach teams were introduced in England in 1999 as per the National Services Framework on Mental Health.^18^ The basic premise was to engage patients with severe mental illness who had failed to engage with generic CMHTs and had high rates of admission.^19^ Despite the international evidence^20^ reporting reduction of in-patient admissions with AOTs, the Randomised Evaluation of Assertive Community Treatment (REACT) in North London^21^ and its follow-up study^22^ did not find any reduction in rates of in-patient admissions in AOT patients compared with usual care by CMHTs. However, rather inexplicably in the UK700 and REACT trails both AOT and CMHT arms showed large reductions in bed usage, which could potentially be because of methodical weaknesses, such as lack of masking. The patients, however, were better engaged and more satisfied with services when under the care of AOTs. Although the results from the UK raise concerns about the continued existence and funding of AOTs, an interesting point raised by Burns^23^ is these studies categorised the AOT as ‘the intervention’ with the control being the usual treatment of being under the care of CMHT. It is difficult to judge the effectiveness of a team based on the sole criterion of reducing in-patient admission despite it being a proxy marker of clinical stability. Also the quality of an individual team can have an impact on the outcome of studies, and there is wide variation in the make-up and fidelity of AOTs.^24^ The dwindling availability of in-patient beds in the UK in recent years has resulted in CMHTs being pragmatic about use of beds in contrast to the USA and Australia.

It could be presumed that the use of CTOs would be high in AOTs as their case-loads include the most disengaged, non-adherent population with associated higher risk levels. Studies comparing patient management in AOTs and CMHTs on the basis of in-patient admissions pre-date the implementation of CTOs in England and Wales. It should also be noted that Australia and North America have had CTOs for decades and few studies have looked at their use by AOTs.

### Findings from this and other studies into the effectiveness of CTOs

A comparative CTO study^25^ from Birmingham conducted soon after introduction of CTO legislation showed that most patients on a CTO tend to be younger males with psychotic illness and from ethnic minority backgrounds. Our sample group showed an overrepresentation of Black males on CTOs. This overrepresentation of Black males has also been found in international studies. Current US census data recorded 21.6% of the North Carolina population as being Black or African American, but 66.2% of patients on a CTO in that state were African American.^26^

Two randomised controlled trials^27,28^ in the USA conducted more than a decade ago failed to show any reduction in bed days between patients on a CTO and controls. One of the trials^28^ in a *post hoc* analysis showed that patients on a CTO after 6 months had fewer admissions to hospital. However, the recent UK-based randomised controlled Oxford Community Treatment Order Evaluation Trial (OCTET),^6^ which compared readmission rates between CTO patients and patients on Section 17 leave, failed to find any difference in readmission rates over a 12-month period and concluded that they could not support the use of CTOs in this country. The findings from the OCTET trial have implications for future policy-making and raise an ethical dilemma regarding curtailment of personal liberties. However, it did not compare the outcomes of CMHT and AOT patients, who are likely to have significant differences both clinically (AOTs use ‘psychosis’ as an admission criterion unlike CMHTs) and in terms of type of interventions. Of note, CHAOS was one of the teams that were included in the OCTET study. The mean clinical contact in the OCTET^6^ study for CTO patients was three times per month. However, level of contact is much higher for CHAOS patients especially in the post-discharge period often for supervised treatment adherence, which may have allowed the development of a more supportive and therapeutic relationship. A well-functioning AOT should be able to provide intensive case management with a high level of psychosocial support, closer supervised medication adherence and monitoring of forensic risks. This is in conjunction with recall to hospital at early stages of disengagement and non-adherence. Delaying recall of non-adherent and disengaged patients by clinicians, before they show significant signs of relapse, is an important factor that potentially negates the effectives of CTOs.^29^ Assertive outreach teams may act earlier in this regard as they tend to have a more risky case-load than CMHTs and are able to detect disengagement earlier because of their level of contact with their patients. Hence, it is possible that the combined ability in delivering intensive case management and earlier recall to hospital by AOTs might make implementation of CTOs more effective, but such individual factors need closer scrutiny.

Assertive outreach teams have been one of the most utilised and funded initiatives to counteract the significant reduction of psychiatric in-patient beds. It has been shown that in the UK, because of the already low usage of in-patient beds, AOTs have not resulted in a reduction in hospital admissions.^30^ The widespread presence of CMHTs, with their ability to provide a degree of intensive case management, also potentially dilutes the clinical benefits of AOTs over generic services. A possibility to consider is that CTOs are most effective in a subpopulation of patients at increased risk, possibly in those with psychotic disorders, irrespective of the make-up of treating teams. In a climate of dwindling mental health funding the relevance of existing AOTs is under close scrutiny. Further evidence looking at their ability to deliver effective CTOs in an expensive and impaired psychiatric subpopulation could decide the future of this model in the UK.

Our study showed a sizable reduction in both overall bed usage and readmission rates following implementation of CTOs. Individual duration of admission was shortened fractionally (by 4 days). However, the total reduction of bed days in the whole cohort was 4529, which contributed to considerable savings for the local healthcare system. The reduction in the number of Mental Health Act assessments involving multiple agencies such as the police and ambulance services possibly reduced costs further as well as reduced patient trauma and stigma. Of note, there was a corresponding 40% reduction in the funding of CHAOS during the study period and so it did not lead to increased service costs. A similar naturalistic study in Suffolk^31^ demonstrated an increase in engagement, a decrease in the number of admissions and increased time spent outside hospital.

In our study the proportion of CTO patients on depot antipsychotics was only 45.9%, which is lower than other studies;^25,29^ this could be because of the ability of the AOT to supervise regular oral medication. Improvement in medication adherence was noted in over three-quarters of the patients during the CTO phase. This could indicate a relationship between reduced hospital admission rate and improved treatment adherence. However, a previous systematic review^10^ had failed to find such a link. Other factors such as improved engagement and reduction of substance misuse may also contribute. Apart from monitoring medication adherence, all patients received input from a dual-diagnosis worker, occupational therapist support, psychoeducation and insight improvement work by their care coordinators. We recognise that in the absence of a dedicated psychologist the amount of psychological input was limited. However, the cover of the CTO provided a framework where these forms of support as well as intensive case management could be delivered to the patient, which is likely to have contributed significantly to the overall clinical outcome.

### Patient views of CTOs

Previous studies have suggested that patients tend to be ambivalent about CTOs, which was echoed in this study. Patients focused on the timely but short admissions to hospital along with viewing CTOs as providing a support framework. Despite the objective evidence of improvement in terms of reduced admissions, improved adherence and reduced drug use, a theme of feeling restricted under a CTO was evident in this cohort. Studies have shown that patients' level of understanding of the impact of CTO legislation and restrictions could be in contrast to their objective clinical improvement.^32^ It has been shown that patients' negative views of CTOs were primarily the result of feeling coerced,^33^ their prolonged use and possibly medication side-effects.^34^ By the nature of their legislative framework, CTOs are ‘expected to be coercive’;^35^ it is important to recognise and address this with psychoeducation and possible peer support.

A qualitative analysis of the use of CTOs in Canada^35^ has shown that initial resentment towards CTOs from patients is superseded by an acceptance of stability on account of the structure of a CTO. Qualitative research on the use of CTOs has shown that patients also see them as providing security, preventing hospital admissions or as an alternative to prison.^36^ Of importance, many patients did not feel that being placed on a CTO affected the therapeutic alliance with their responsible clinician.^33^ However, these findings could be the result of systematic bias as patients who agree to be involved in such studies could be assumed to have developed greater insight.

### Care coordinators' views of CTOs

The feedback we obtained from the care coordinators generally reflected a positive view of CTOs, which were seen to support adherence, reduce hospital admissions and provide therapeutic structure. The care coordinators also raised some pertinent questions about the ethics of subjecting patients to restrictions in the community and the increased administrative workload on clinicians. These are recurrent themes that have been raised by clinicians in other studies^35^ despite generally viewing CTOs as a positive step and in the long-term helping form a therapeutic alliance.^37^

An ethical debate about CTOs is beyond the remit of this article as decision-making around implementation is complex. The political and media fervour of highlighting acts of violence by psychiatric patients could lead to CTOs being unjustifiably used as a risk management tool.^34^ The motivations behind the decision to subject a patient to a CTO needs to be explicitly clinical and should be in the best interest of the patient at an individual level and not just for cost-benefits or simply as a risk management tool, although these are important considerations at a public health level.

### Strengths and limitations

The main strengths of the paper include the sizable number of patients studied under one team with the patients acting as their own comparators in a naturalistic setting. The quality of the data was of a high standard as they were obtained from the Trust electronic patient database. The retrospective nature of the study also negates interventional bias. The main weakness of the study was the absence of a non-CTO comparison group to measure any changes in service provision on clinical outcome measures. The study was based in an inner-London borough and involves only one individual AOT and may not be generalisable to other settings.

### Implications

Despite the conflicting evidence of the benefits of CTOs, their use has become widespread in England and Wales. This study indicates the potential benefits of CTOs in reducing readmissions and bed occupancy in a disengaged AOT population, possibly through improved engagement, adherence to treatment and reduced substance misuse. This may have significant clinical, service planning and cost-benefit implications that need further research. Any such gains need to be balanced with the potential negative impact on patients' autonomy, therapeutic alliance as well as possible deskilling of staff as a result of reliance on coercion rather than interactive therapeutic approaches to achieve adherence,^38^ excessive medicalisation of care through depot use^39^ and the extra logistical burden on medical staff.^40^ However, CTOs cannot be ignored as a potential therapeutic tool in supporting adherence in a difficult-to-engage and potentially high-risk patient group. At its best, it can be helpful in allowing patients to remain stable in the community, build therapeutic relationships with clinicians and reduce the cost burden on health and criminal justice systems.
